# Comprehensive Analysis of the Effects of Genetic Ancestry and Genetic Characteristics on the Clinical Evolution of Oral Squamous Cell Carcinoma

**DOI:** 10.3389/fcell.2021.678464

**Published:** 2021-12-07

**Authors:** Junfeng Guo, Xiaoping Liu, Yi Zeng, Taotao Liang, Kanglai Tang, Junfeng Guo, Weiwei Zheng

**Affiliations:** ^1^ Sports Medicine Center, Southwest Hospital, Third Military Medical University, Chongqing, China; ^2^ Department of Stomatology, The 970th Hospital of the Joint Logistics Support Force, Yantai, China; ^3^ Department of Stomatology, Southwest Hospital, Third Military Medical University, Chongqing, China

**Keywords:** OSCC, genetic ancestry, genomic characteristics, TCGA, prognosis

## Abstract

Oral squamous cell carcinoma (OSCC), a kind of malignant cancer, is associated with increasing morbidity and mortality. Patients with different genetic ancestries may respond differently to clinical treatment. The limited understanding of the influence of genetic ancestry and genetic characteristics on OSCC impedes the development of precision medicine. To provide a reference for clinical treatment, this study comprehensively analyzed multigenomic differences in OSCC patients with different genetic ancestries and their impact on prognosis. An analysis of data from OSCC patients with different genetic ancestries in The Cancer Genome Atlas (TCGA) showed that the overall survival (OS) of African (AFR) patients was lower than that of primarily European (EUR) patients, and differences were also observed in the tumor–stroma ratio (TSR) and tumor-infiltrating lymphocytes (TILs), which are associated with prognosis. *FAT1* is a key mutant gene in OSCC, and it has inconsistent effects on clinical evolution for patients with diverse genetic characteristics. *PIKfyve* and *CAPN9* showed a significant difference in mutation frequency between EUR and AFR; *PIKfyve* was related to Ki-67 expression, suggesting that it could promote tumor proliferation, and *CAPN9* was related to the expression of Bcl-2, promoting tumor cell apoptosis. A variant methylation locus, cg20469139, was correlated with the levels of PD-L1 and Caspase-7 and modulated tumor cell apoptosis. A novel ceRNA model was constructed based on genetic ancestries, and it could accurately evaluate patient prognosis. More importantly, although T cell dysfunction scores could determine the potential of tumor immune escape, the efficacy was obviously affected by patients’ genetic ancestries. To provide patients with more precise, personalized therapy and to further improve their quality of life and 5-year survival rate, the influence of genetic ancestry should be fully considered when selecting treatments.

## Introduction

Oral squamous cell carcinoma (OSCC) is one of the most common malignant tumors in the oral cavity or head and neck region and is listed as the eighth most common cancer type in the world (Carnielli et al., 2018; Wang et al., 2021), accounting for approximately 90% of oral tumors and 3% of systemic malignant tumors (Zhao et al., 2020; Lu et al., 2021). According to the latest Global Cancer Incidence, the GLOBOCAN 2020 estimates of cancer incidence and mortality produced by the International Agency for Research on Cancer (IARC), newly developed OSCC originating from the alveolar ridge, buccal mucosa, bottom of the oral cavity, upper jaw, tongue, and other parts of the mouth accounted for 377,713 cases worldwide, with a death toll of 177,757 (Sung et al., 2021), which is much higher than the 300,000 new cases and 145,000 deaths reported globally in 2012 (Ferlay et al., 2015). The morbidity and mortality of OSCC are increasing year by year, and the 5-year survival rate of OSCC continues to be lower than 60% (Ho et al., 2019; Bosetti et al., 2020; Ferreira et al., 2021). Even for patients who received standard therapy with proper combinations of surgical and nonsurgical treatments, the recurrence rate of OSCC was still relatively high in the range of 18–76% (da Silva et al., 2012; Wang et al., 2020). Therefore, identifying new treatment methods and more comprehensive prognostic indicators is particularly important.

**FIGURE 1:**
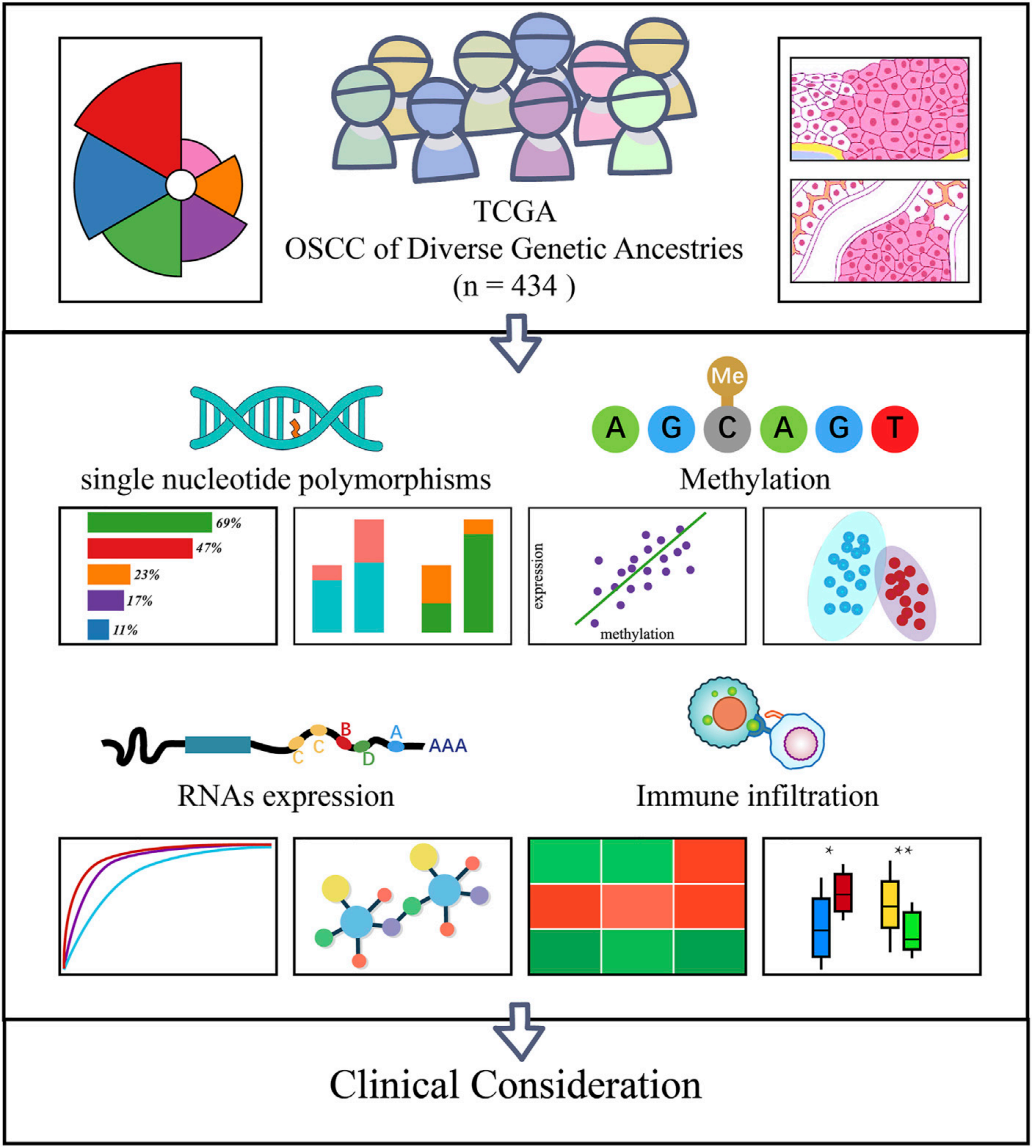
Flowchart showing an overview of our study design.

People of different genetic ancestries often show different reproductive genetic characteristics (Price et al., 2006; Huynh-Le et al., 2021; Zhang et al., 2021). Due to exposure to different living environments or different pathogenic or nonpathogenic factors, their tumor morbidities, outcomes, prognoses, and pathogenic molecular characteristics differ (Yang et al., 2011; Vineis and Fecht, 2018; Ou et al., 2020). Previous studies have mainly focused on molecular differences between normal oral tissues and tumor tissues and their effects (Chen et al., 2020; Yin et al., 2020). No report is available on differences in molecular-genomic characteristics of OSCC patients of different genetic ancestries. In this study, The Cancer Genome Atlas (TCGA) was used as the data source. We intend to use a comprehensive cross-platform and multigenomic analysis, including hematoxylin–eosin (HE) staining, somatic mutations, methylation, RNA expression, immune infiltration, and immune response data, to analyze the differences in the prognoses of OSCC patients of various genetic ancestries, especially primarily European (EUR) and African (AFR). We sought to exploit these results to improve our understanding of the molecular and cellular effects of ancestry across clinical evolution and the relationship between ancestry and clinical treatment and prognosis to provide new options for the effective precision treatment of OSCC patients (Figure 1).

## Materials and Methods

### Data Collection

The hematoxylin–eosin (HE) staining results, single-nucleotide variant data, methylation data, RNA sequencing data, and clinical data of OSCC patients were downloaded from the TCGA database (https://cancergenome.nih.gov/). The genetic ancestry–related data of OSCC patients were obtained from Carrot-Zhang et al. (2020). In addition, the reverse-phase protein array data of patients were downloaded from The Cancer Proteome Atlas (TCPA) database (https://www.tcpaportal.org/tcpa/) (Li et al., 2013). T cell dysfunction scores of OSCC patients were downloaded from the Tumor Immune Dysfunction and Exclusion (TIDE) database (http://tide.dfci.harvard.edu) (Fu et al., 2020).

### Pathological and Survival Analyses of Patients With Different Genetic Ancestries

Based on HE staining results, we evaluated the pathological differentiation degree (well, moderately, poorly) of OSCC patients and the tumor–stroma ratio (TSR) according to the method reported in the literature, which defines a TSR ≥50% as high and a TSR <50% as low (van Pelt et al., 2018; Hagenaars et al., 2021). Tumor-infiltrating lymphocytes (TILs) were also evaluated. TILs ≥10% was considered high, and TILs <10% was considered low (Salgado et al., 2015; Veatch et al., 2021). Differences in the TSR and TILs between OSCC patients of different genetic ancestries were analyzed. In a Kaplan-Meier survival analysis on primarily European (EUR) and African (AFR) OSCC patients, to eliminate the effects of old age and short follow-up times, OSCC patients older than 80 years and those with a follow-up time less than 90 days were excluded.

### Analysis of Somatic Mutations and Methylation

We analyzed differences in single-nucleotide variant frequencies between OSCC patients with different genetic ancestries in the TCGA database. Kaplan-Meier survival analysis was performed to compare the effects of mutations between EUR and AFR OSCC patients. Then, we used the cBioPortal database (http://www.cbioportal.org/) (Gao et al., 2013) to perform and visualize a pancancer statistical analysis of *FAT1* mutations.

The difference in locus methylation between EUR and AFR OSCC patients was analyzed, where | log2FC | > 1 and a false discovery rate (FDR) < 0.05 were the thresholds for differential methylation. We performed a principal component analysis (PCA) on the differentially methylated sites to determine whether they could effectively differentiate between EUR and AFR OSCC patients. At the same time, survival-related methylation sites were identified, and a correlation analysis between these sites and protein levels was conducted to explore the functions of survival-related methylation sites.

### Construction of a ceRNA Risk Regression Model

We, respectively constructed mRNA, lncRNA, and miRNA risk regression models of OSCC patients. Kaplan-Meier survival analysis, multiclinical information heat maps, and receiver operator characteristic (ROC) curves were used to evaluate the effects of the three models. We integrated the three models, yielding the ceRNA risk regression model, and compared it with the three component models.

An independent prognostic analysis was conducted to assess the effects of genetic ancestry and the risk scores from the ceRNA models on the prognosis of OSCC patients. The ceRNA risk regression model was also applied to the pancancer analysis to determine whether it had specificity for OSCC. Finally, we constructed the ceRNA network of mRNA-miRNA-lncRNA to further investigate the relationship between RNAs. We also used the STRING database (http://string-db.org/cgi/input.pl) (Szklarczyk et al., 2019) to construct the protein interaction network for genetic ancestry.

### Analysis of Immune Cell Infiltration

We used the CIBERSORTx algorithm (Newman et al., 2019) to calculate the infiltration of 22 kinds of immune cells in OSCC patients and analyzed differences in the immune cell distribution between patients of different genetic ancestries. We compared the effect of using T cell dysfunction scores to evaluate prognoses between OSCC patient groups of different genetic ancestries.

### Statistical Analysis

Bilateral tests were performed for all statistical tests. A *p* value less than 0.05 was considered statistically significant. R software version 4.0.0 (https://www.r-project.org/) was used for analyses. Some R packages were used in this study, including “limma,” “RColorBrewe,” “ggplot2,” “survival,” “maftools,” “corrplot,” “pheatmap,” “survivalROC,” “reshape2,” and “forestplot.” The ceRNA network was visualized using Cytoscape_3.7.2 (https://cytoscape.org) (Shannon, 2003).

## Results

### OSCC Patients and Their Genetic Ancestry Distributions

**FIGURE 2:**
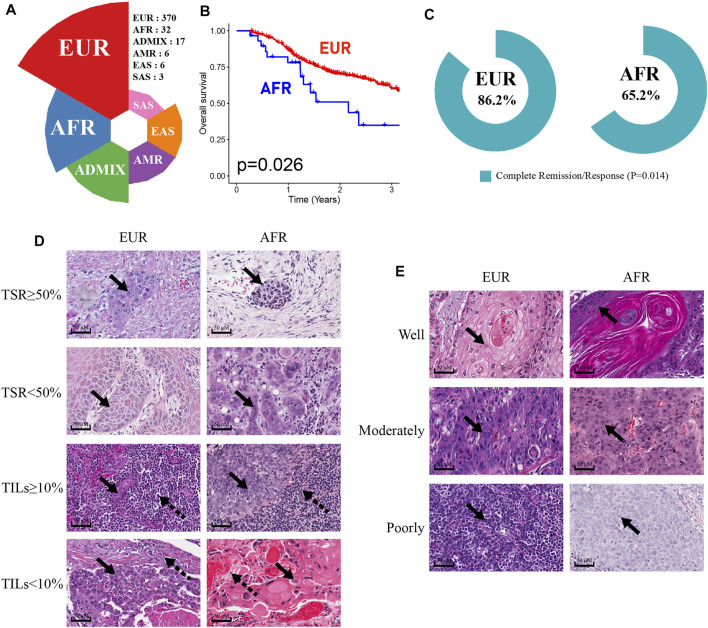
Characteristics of OSCC patients of EUR or AFR genetic ancestry. **(A)** The percentage of OSCC patients with different genetic ancestries in the TCGA database. **(B)** The OS of patients of AFR ancestry was significantly lower than that of patients of EUR ancestry. **(C)** The complete remission rates of patients of EUR and AFR ancestries following initial treatment were significantly different. **(D)** The results of HE staining were used to divide EUR and AFR ancestry patients into TSR ≥50% and TSR <50% groups and TILs ≥10% and TILs <10% groups (scale bar: 50 μM, magnification: 40X; the solid arrowhead indicates tumor cells, and the dotted arrowhead indicates lymphocytes). **(E)** HE staining results were used to divide EUR and AFR ancestry patients into well differentiated, moderately differentiated, and poorly differentiated tumor groups (scale bar: 50 μM, magnification: 40X; the solid arrowhead indicates tumor cells).

We analyzed the genetic ancestries of OSCC patients in the TCGA database. Among 434 patients, primarily European ancestry (EUR; *n* = 370) accounted for the highest proportion at 85.25%, African ancestry (AFR; *n* = 32) accounted for 7.37%, Native/Latin American ancestry (AMR; *n* = 6) accounted for 1.38%, East Asian ancestry (EAS; *n* = 6) accounted for 1.38%, South Asian ancestry (SAS; *n* = 3) accounted for 0.69%, and admixed descent ancestry (ADMIX; *n* = 17) accounted for 3.92% of the sample (Figure 2A). As more than two-thirds of TCGA donors were from the United States (Carrot-Zhang et al., 2020), the numbers of OSCC patients with AMR, EAS, SAS, and ADMIX genetic ancestries in the database were relatively small and may not accurately represent the corresponding overall OSCC population. Therefore, we only discuss differences between the EUR and AFR.

First, the Kaplan-Meier survival analysis indicated that the overall survival (OS) of AFR patients was significantly lower than that of EUR patients (*p* = 0.026) (Figure 2B). When we compared the initial treatment responses of EUR and AFR OSCC patients, the percentage of complete remission/complete response (CR) in EUR was significantly higher than that in AFR. The CR rates of EUR and AFR OSCC patients were 86.2 and 65.2%, respectively (*p* = 0.014) (Figure 2C). Analysis of the HE staining results showed that the TSR and TILs in EUR and AFR OSCC patients were significantly different (Figure 2D; Table 1) (*p* < 0.05), but the pathological differentiation was not significantly different between the two groups of patients (Figure 2E; Table 1).

**TABLE 1:** Differential analysis of the differentiation degree, the TSR, and TILs in OSCC patients of EUR and AFR ancestries (53 cases without corresponding data among patients of EUR ancestry and 1 case without corresponding data among patients of AFR ancestry).

Variable	Ancestry (no. Of patients)		*p* Value
	EUR (*n* = 317)	AFR (*n* = 31)	
Differentiation			0.933
Well	242	24	
Moderately	58	5	
Poorly	17	2	
Tumor Stroma Ratio			0.028
TSR≥50%	52	10	
TSR<50%	265	21	
Tumor Infiltrating Lymphocytes			0.009
TILs≥10%	147	22	
TILs<10%	170	9	

### Somatic Mutation Differences Between OSCC Patients With Different Genetic Ancestries

**FIGURE 3:**
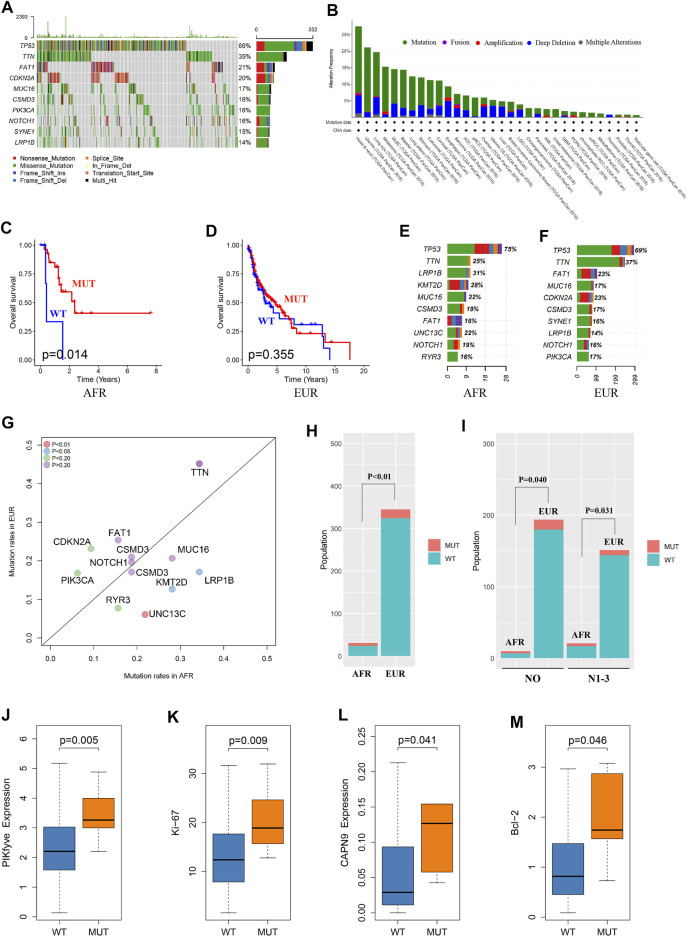
Somatic mutation differences between EUR and AFR OSCC patients. **(A)** Waterfall plots of the genes with the top 10 single-nucleotide variant frequencies among all OSCC patients in the TCGA database. **(B)** The pancancer mutation frequency of *FAT1*, which shows that the mutation frequency was highest in head and neck squamous cell carcinoma. **(C)** The survival of the *FAT1*-mutated patients was significantly better than that of the wild-type patients in the AFR ancestry. **(D)** No significant difference in survival was found between *FAT1*-mutated and wild-type patients in the EUR ancestry. **(E)** The genes with the top 10 mutation frequencies in the AFR ancestry. **(F)** The genes with the top 10 mutation frequencies in the EUR ancestry. **(G)** Differences in the genes with the highest mutation frequencies between EUR and AFR ancestries. **(H)** The mutation frequency of *UNC13C* was significantly different between the EUR and AFR ancestries. **(I)** The mutation frequency of *UNC13C* in N0 and in N1-3 stages was significantly different between the EUR and AFR ancestries. **(J)** *PIKfyve* mutation caused increased expression of this gene. **(K)** OSCC patients with *PIKfyve* mutation had higher levels of Ki-67 expression. **(L)** *CAPN9* mutation caused increased expression of this gene. **(M)** OSCC patients with *CAPN9* mutation had higher Bcl-2 expression.

We analyzed somatic mutations in OSCC patients in the TCGA database. The mutation frequency of *FAT1* in OSCC patients was 21%, rendering it the third-most often mutated gene in this patient set (Figure 3A). We performed a pancancer analysis of *FAT1* mutations. As shown in Figure 3B, *FAT1* had the highest mutation frequency in head and neck squamous cell carcinoma, suggesting that *FAT1* mutation might play an important role in the occurrence and development of OSCC (Nishikawa et al., 2011; Hayes et al., 2016; Hsu et al., 2019; Kuo et al., 2019). We analyzed the effect of *FAT1* mutation on the survival of patients of EUR and AFR. The results showed that among patients of AFR, the OS of *FAT1*-mutated patients was significantly better than that of wild-type patients (Figure 3C), but this difference was not significant in EUR (Figure 3D). We next compared genes with the top 10 mutation frequencies in AFR and EUR (Figures 3E–G). UNC13C only appeared in the top 10 genes of AFR and showed a significant difference in mutation frequency between the EUR and AFR (*p* < 0.01) (Figure 3H). Considering the impact of clinical stage, we also obtained the same results in N0 and N1-3 patients, respectively (Figure 3I). Among the other differentially mutated genes, the mutation frequency of *PIKfyve* in AFR was significantly higher than that in EUR (AFR = 9.38%, EUR = 1.27%, *p* < 0.05). PIKfyve mutations mainly lead to an increased expression of the same gene (Figure 3J). Hou et al. (2018) showed that *PIKfyve* can promote tumor cell proliferation. Therefore, we analyzed the level of Ki-67, a cell proliferation–related marker, and found that the Ki-67 level was significantly higher in patients with *PIKfyve* mutations (Figure 3K), suggesting that *PIKfyve* mutation promoted tumor proliferation. The mutation frequency of *CAPN9* in AFR was also significantly higher than that in EUR (AFR = 6.25%, EUR = 0.75%, *p* < 0.05), and *CAPN9* mutations were associated with higher expression of the same gene (Figure 3L). Peng et al. (2016) found that overexpression of *CAPN9* promoted tumor cell apoptosis. We analyzed the level of Bcl-2, an apoptosis-related marker, and found that Bcl-2 was significantly elevated in patients with *CAPN9* mutations (Figure 3M), suggesting that *CAPN9* mutations promote tumor cell apoptosis by increasing this gene’s expression, thus playing a role in cancer inhibition.

### DNA Methylation Differences Between OSCC Patients With Different Genetic Ancestries

**FIGURE 4:**
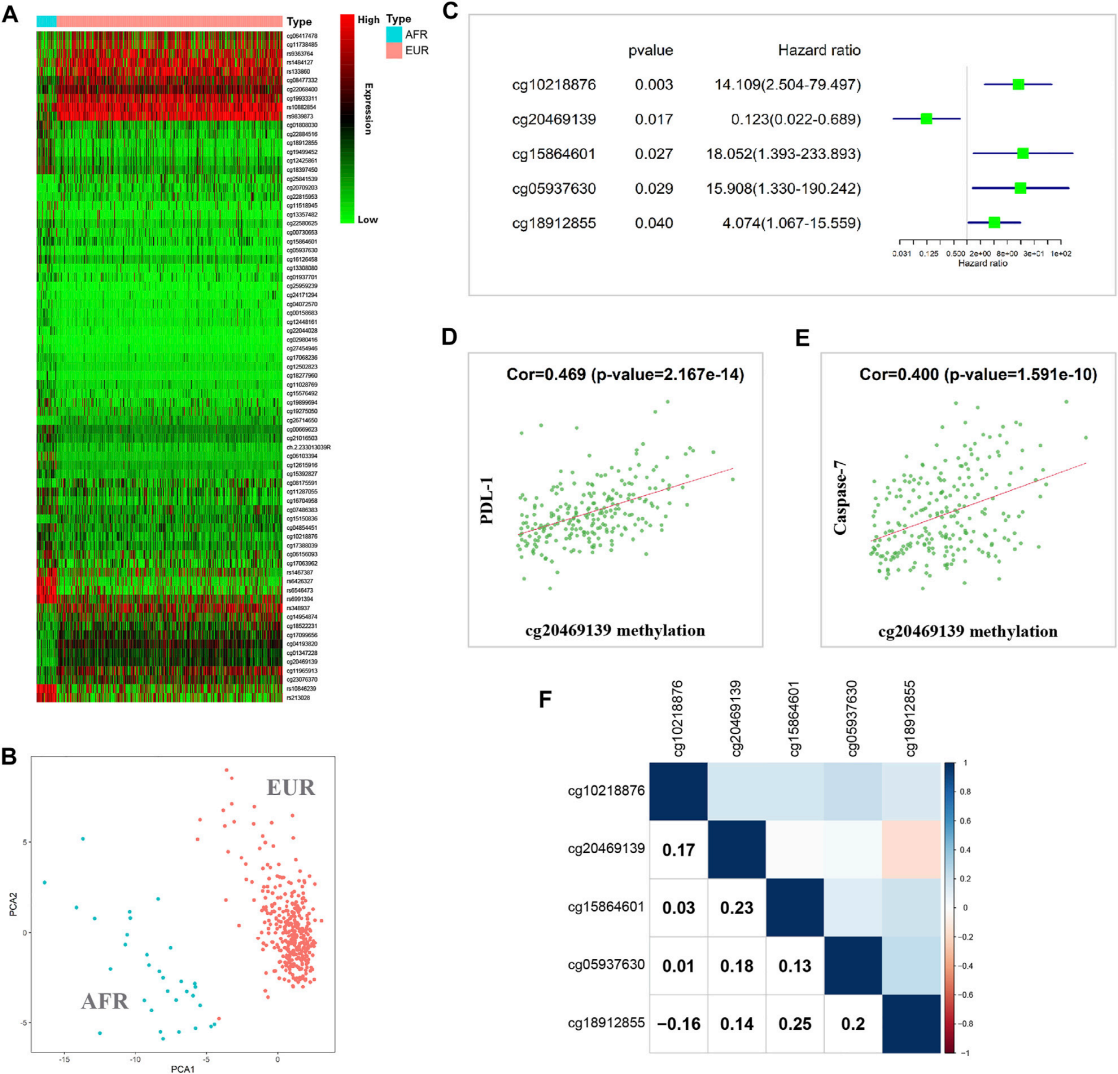
Methylation differences between EUR and AFR OSCC patients. **(A)** Heat map of differentially methylated sites between the EUR and AFR ancestries. **(B)** Differentially methylated sites can effectively differentiate patients of EUR and AFR ancestries. **(C)** Five differentially methylated sites were associated with the prognosis of OSCC patients. **(D)** The degree of methylation of the cg20469139 site was significantly correlated with the PDL-1 level. **(E)** The degree of methylation at the cg20469139 site was significantly correlated with the caspase-7 level. **(F)** Correlations between the methylation levels of the five survival-related loci.

When the methylation information of OSCC patients in the TCGA database was analyzed, the results showed that 75 methylated sites significantly differed between EUR and AFR (Figure 4A). PCA showed that these sites could effectively differentiate patients of EUR and AFR (Figure 4B). Univariate Cox analysis showed that five methylation sites were associated with the prognosis of OSCC patients (Figure 4C). Correlation analysis between the methylation data of these sites and the protein expression data in the TCPA database showed that the degree of methylation at site cg20469139 was correlated with the levels of two apoptosis-related proteins, PDL-1 (Figure 4D) and caspase-7 (Figure 4E), suggesting that this site may affect the prognosis of patients by regulating tumor cell apoptosis.

We further performed a correlation analysis of the five methylation sites, and the results showed that the correlation between these methylation sites was relatively low, indicating that the information carried by these sites had almost no redundancy or crossover; therefore, these sites might affect tumor progression in different manners (Figure 4F). In addition, we found that cg10218876 was located in the *TNNT1* gene. TNNT1 promotes the proliferation of breast cancer cells by promoting the G1/S transition (Shi et al., 2018). cg20469139 is located in the *RNF135* gene. The RNF135 protein has been reported to inhibit the proliferation of tongue squamous carcinoma cell line SCC25 and participate in PTEN signaling transduction (Jin et al., 2016). cg15864601 is located in the *C17orf97* gene. Coexpression of CK20 (also known as C17orf97) and Ki-67 may play an important role in the progression of bladder cancer and can be used as a prognostic indicator (Ye et al., 2010). cg05937630 is located in the *AKR7A3* gene. AKR7A3 inhibits the tumorigenicity and chemoresistance of hepatic carcinoma by attenuating ERK, c-Jun, and NF-κB signaling pathways (Chow et al., 2017). cg18912855 is located in the *PACS2* gene. PACS2 can promote ErbB signal transduction by regulating the recovery of the metalloproteinase ADAM17, thus affecting the biological behavior of tumors (Dombernowsky et al., 2015).

### ceRNA Expression Differences Between OSCC Patients With Different Genetic Ancestries

**FIGURE 5:**
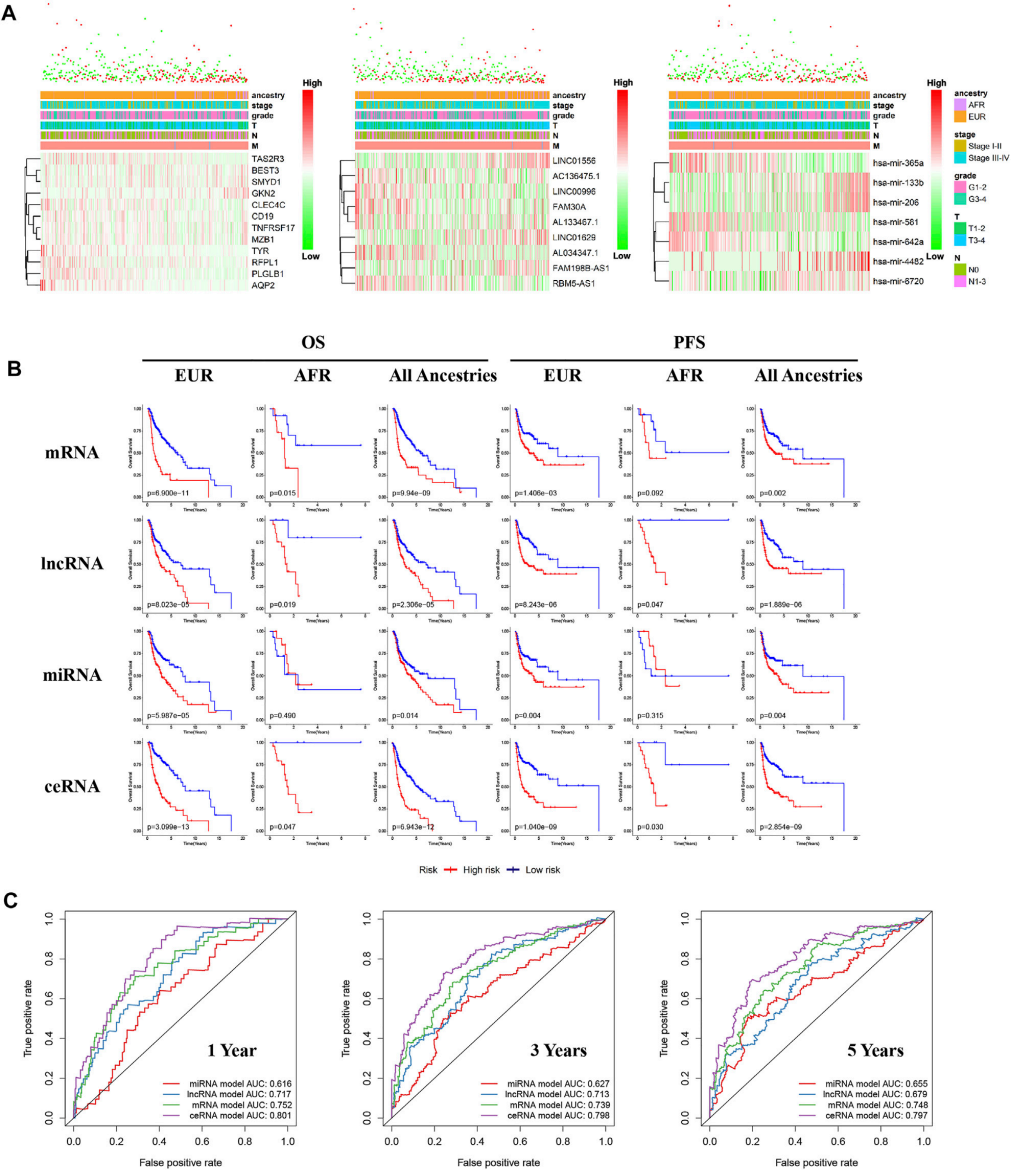
Differences in ceRNA expression between EUR and AFR OSCC patients. **(A)** The multiclinical trait heat map of the mRNA, lncRNA, and miRNA risk regression models (from left to right). **(B)** The mRNA, lncRNA, miRNA, and ceRNA risk regression models were used to predict the prognoses of OSCC patients of EUR, AFR, and all genetic ancestries. **(C)** ROC curves for the prognosis at 1, 3, and 5 years from the four risk regression models.

We performed differential expression analyses of mRNA, lncRNA, and miRNA between OSCC patients of different genetic ancestries and constructed respective risk regression models based on differentially expressed mRNA, differentially expressed lncRNA, and differentially expressed miRNA. From the risk heat map, we found that as the risk value increased, the proportion of deaths significantly increased, and the survival time also decreased (Figure 5A). The results of the Kaplan-Meier survival analysis showed that all three risk regression models accurately assessed the prognoses of OSCC patients of all genetic ancestries, including EUR and AFR. The OS and progression-free survival (PFS) of the low-risk group were superior to those of the high-risk group (Figure 5B).

**FIGURE 6:**
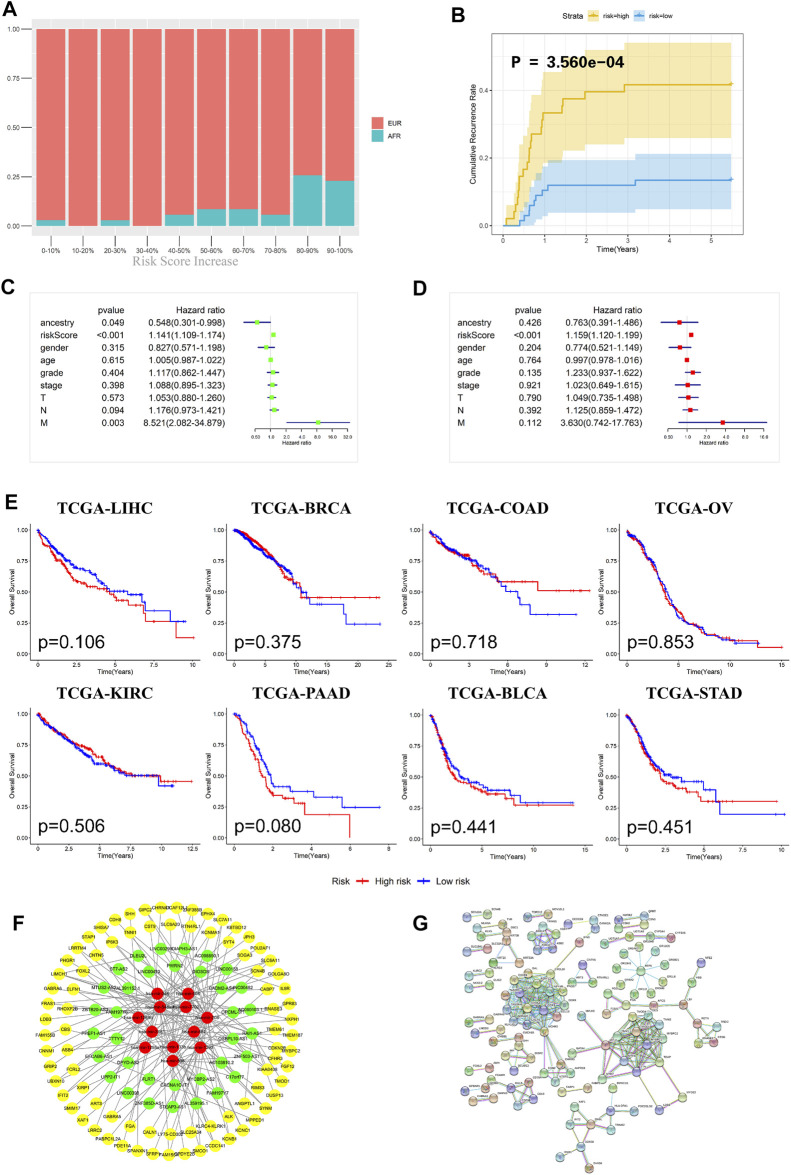
Evaluation effectiveness and specificity of the ceRNA risk regression model. **(A)** The percentages of EUR and AFR OSCC patients in different risk stratification groups. **(B)** In the ceRNA risk regression model, the relapse rate of the OSCC patients in the high-risk group was significantly higher than that in the low-risk group. **(C)** Univariate Cox analysis of the ceRNA risk regression model and related clinical information such as genetic ancestry. **(D)** Multivariate Cox analysis of the ceRNA risk regression model and related clinical information such as genetic ancestry. **(E)** No significant difference in the prognoses of all cancer patients was found when using the ceRNA risk regression model. **(F)** The ceRNA network of differentially expressed RNAs between EUR and AFR ancestries. **(G)** The interaction network of differentially expressed proteins between the EUR and AFR ancestries.

To further evaluate the prognosis of OSCC patients, we integrated the three models to establish a ceRNA risk regression model. From the ROC curve (Figure 5C), we found that the predictive abilities of the ceRNA risk regression model for the prognoses of OSCC patients at one, three, and 5 years were all better than those of the mRNA, lncRNA, and miRNA risk regression models alone, indicating a satisfied predictive effect. Based on the risk score calculated by the ceRNA model, the patients were divided into 10 groups from low to high. As shown in Figure 6A, with the risk score increased, the proportion of patients of AFR increased, and their prognoses were poor, which is consistent with the results of the Kaplan-Meier survival analysis showing that the OS of patients of AFR was significantly lower than that of patients of EUR.

Recurrence is an important factor affecting the survival of OSCC patients. The recurrence plot (Figure 6B) showed that the relapse rate was significantly higher in the high-risk patients than in the low-risk patients (*p* < 0.01), indicating that the ceRNA risk regression model can not only predict the survival of patients but also accurately predict the risk of tumor recurrence. To further evaluate the effects of different genetic ancestries on prognosis, we performed an independent prognostic analysis. Univariate Cox analysis suggested that genetic ancestry and ceRNA risk scores were correlated with prognosis (Figure 6C). However, multivariate Cox analysis showed that only risk scores could be used as an independent prognostic factor for OSCC patients (Figure 6D).

We have used this ceRNA risk regression model in pancancer prognostic evaluations and found that it could not accurately assess the prognosis of patients with other cancers (Figure 6E), indicating that this model has strong specificity for OSCC patients.

Lastly, we constructed a ceRNA and protein interaction network to analyze interactions between differentially expressed RNAs in OSCC patients with different genetic ancestries. As shown in Figures 6F,G, these mRNAs, lncRNAs, and miRNAs had extensive and close associations.

### Immunity Differences Between OSCC Patients With Different Genetic Ancestries

**FIGURE 7:**
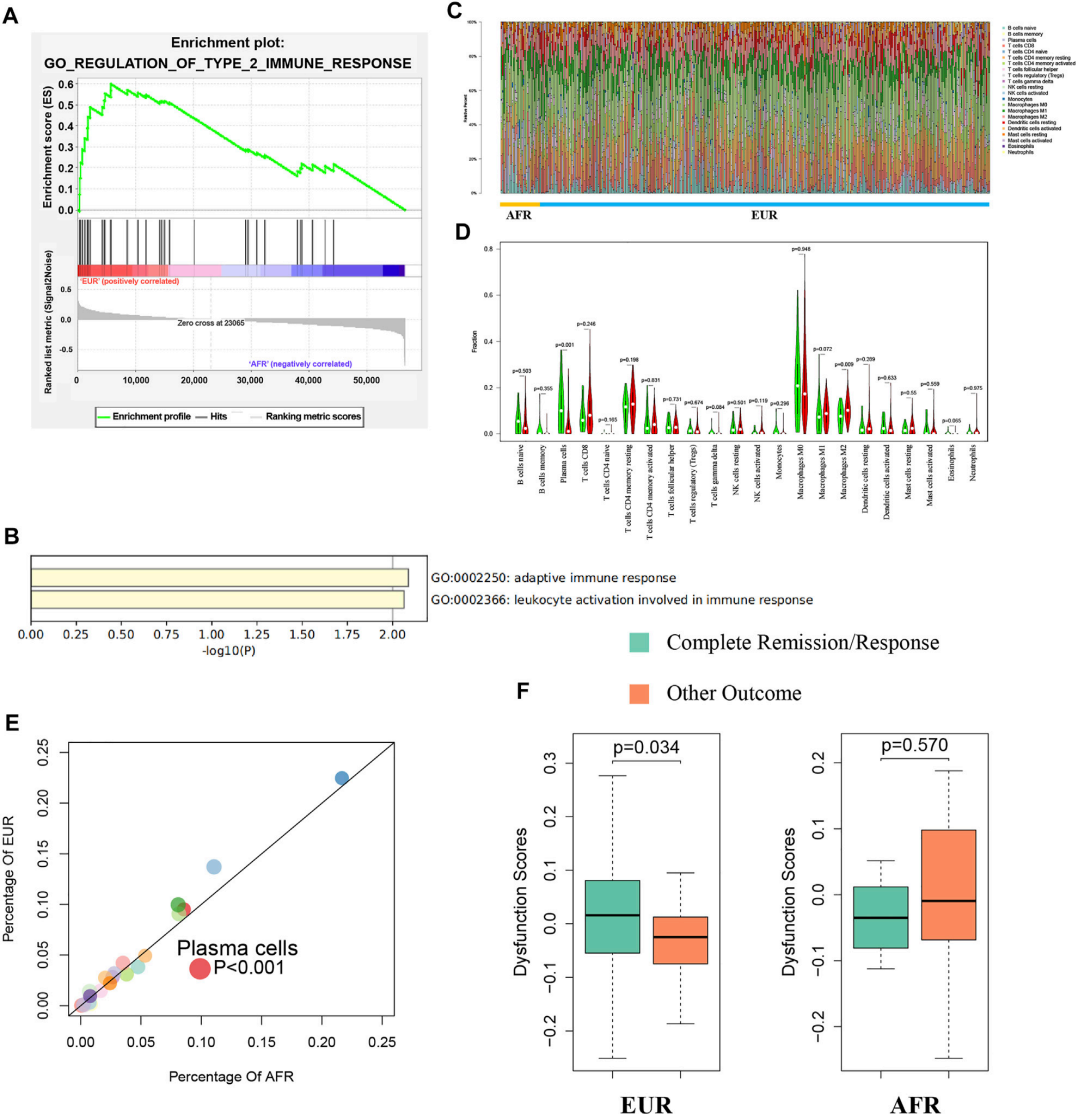
Differences in immunity between EUR and AFR OSCC patients. **(A)** GSEA of differentially expressed genes between EUR and AFR ancestries in the GO_REGULATION_OF_TYPE_2_IMMUNE_RESPONSE pathway. **(B)** Enrichment analysis of genes used to build the ceRNA risk regression model using Metascape showed that these genes were associated with the immune response. **(C)** Histogram of the degree of infiltration of different immune cell types in EUR and AFR ancestries. **(D)** Violin plot of the differences in the degree of infiltration of different immune cell types between EUR and AFR ancestries. **(E)** Scatter plot of the differences in the degree of infiltration of different immune cell types between EUR and AFR ancestries. **(F)** Relationships between T cell dysfunction scores and preliminary treatment outcomes in EUR and AFR OSCC patients.

We used gene set enrichment analysis (GSEA) to perform an enrichment analysis on the differentially expressed genes between EUR and AFR, and found that they were significantly enriched in the GO_REGULATION_OF_TYPE_2_IMMUNE_RESPONSE pathway (Figure 7A). Metascape was used to perform an enrichment analysis on the genes used to build the ceRNA risk regression model (Figure 7B), which also showed that these genes were associated with the immune response. Therefore, we speculated that OSCC patients with different genetic ancestries have differences in immune infiltration and immune response, which affected their prognoses.

We used the CIBERSORTx algorithm to calculate the infiltration levels of 22 immune cells in EUR and AFR (Figure 7C). The violin plot (Figure 7D) and the scatter plot (Figure 7E) showed that the degree of plasma cell infiltration in EUR was significantly higher than that in AFR (*p* < 0.01). Gentles et al. (2015) showed that the plasma cell content is a significant predictor of the survival of patients with solid tumors, which is consistent with our finding that the prognosis of EUR OSCC patients was better than that of AFR. In EUR, the T cell dysfunction scores of patients with complete remission after initial treatment was significantly higher than that of patients with incomplete remission (*p* < 0.05); however, the difference between the two patient groups was not significant in the population with AFR (Figure 7F).

**FIGURE 8:**
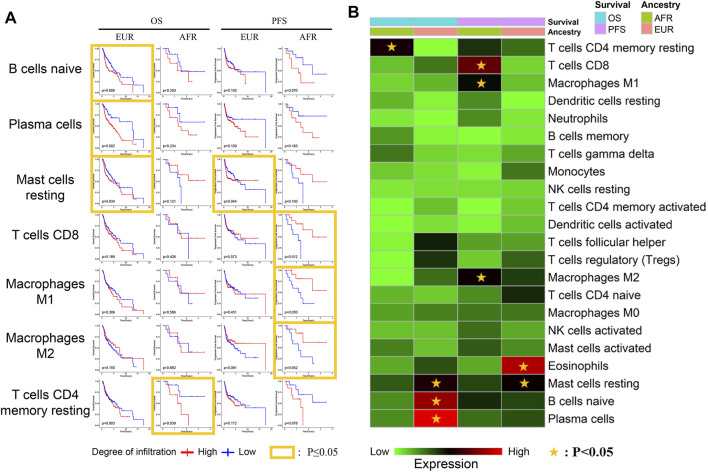
Relationship between immune cells and survival with different genetic ancestries. **(A)** The relationship between immune cell infiltration and survival in EUR and AFR OSCC patients. **(B)** P-value heat map of the relationship between immune cell infiltration and survival in EUR and AFR OSCC patients.

We further explored the relationship between the infiltration of different immune cells and survival. Naive B cells, plasma cells, and mast cells were associated with the OS of EUR but not AFR. CD8^+^ T cells, M1 macrophages, and M2 macrophages were associated with the PFS of AFR but not EUR, further confirming the differences in immune infiltration between the patients of the two genetic ancestries (Figures 8A,B).

## Discussion

OSCC refers to a group of heterogeneous tumors originating from the mucosal lining (Meng et al., 2021). Most of these tumors are associated with lifestyle factors, such as smoking, excessive drinking, and betel nut chewing (Adil et al., 2021; Weiße et al., 2021). Cetuximab, which targets epidermal growth factor receptors, was approved for OSCC treatment in 2006 and remains the only available targeted molecular therapy for OSCC to date (Chai et al., 2020; Johnson et al., 2020). Therefore, exploring more effective targets and accurately assessing the prognosis are key to improving the 5-year survival rate of OSCC patients.

With the rapid development of precision medicine, a wide range of personal data, including clinical, lifestyle, genetic, and biological marker information, must be fully considered when selecting treatments (Konig et al., 2017; Elmore et al., 2021). Different genetic ancestries play important roles in the occurrence and progression of tumors. For example, Jiagge et al. (2018) found that the mortality rate of breast cancer among African-American women was significantly higher than that among white American women. Gentles et al. (2015) showed that some genomic differences may lead to higher prostate cancer morbidity and mortality in African-American men. In this study, we comprehensively analyzed the molecular characteristics of OSCC in patients between EUR and AFR. Our results showed that OSCC patients with different genetic ancestries had significant differences (*p* < 0.05) not only in initial treatment responses and overall survival but also in the TSR and TILs, two variables that affect prognosis.

At the molecular level, somatic mutations in genes such as *FAT1* are more common in OSCC than in other tumors (Chai et al., 2020; Sequeira et al., 2020). We found that *FAT1* mutation had different effects on prognosis in OSCC patients with different genetic ancestries. In addition, the mutation frequencies of *UNC13C*, *PIKfyve*, and *CAPN9* were significantly different between EUR and AFR (*p* < 0.05) and might affect prognosis by regulating tumor proliferation and apoptosis. Five methylation sites differed between OSCC patients with different genetic ancestries and affected their prognoses (*p* < 0.05). Our ceRNA risk regression model revealed differences in survival and prognosis between AFR and EUR OSCC patients (*p* < 0.05). The degree of plasma cell infiltration in EUR was significantly higher than that in AFR (*p* < 0.05). The prognosis of EUR was better than that of AFR, which is consistent with the results of Gentles et al. (2015). Jiang et al. (2018) found that T cell dysfunction scores could predict the responses of patients to immunotherapy. We found differences in complete remission and incomplete remission between OSCC patients with different genetic ancestries, suggesting that the predictive ability of T cell dysfunction scores might be different in patients with different genetic ancestries and that the effects of different genetic ancestries should be considered when using T cell dysfunction scores.

The present study has some limitations. TCGA cohort consists of convenience samples, so it may not represent a general cancer population (Wang et al., 2018). Only almost 15% OSCC patients in our study were at least partially non-EUR, a robust distinction of tumor-specific ancestral associations will require comparative analyses of more tissue samples from patients of different genetic ancestries, especially non-EUR OSCC patient tissues. However, we found fairly strong evidence suggesting that genetic ancestry–based differences in somatic mutations, methylation, ceRNA expression, immune infiltration, and immune responses are associated with cancer prognosis. In particular, the differences in OSCC variables between the EUR and AFR populations suggest that genetic ancestry should be considered when contemplating disease-causing factors and related treatments. Our ceRNA risk regression model can be used to evaluate the prognosis of OSCC patients with different genetic ancestries and is specific for OSCC. These results provide new ideas for the treatment and prevention of OSCC in patients with different genetic ancestries.

This study explored the effect of genetic ancestry on the clinical evolution of OSCC patients and the molecular mechanism through a comprehensive analysis of multigenomic differences between OSCC patients with different genetic ancestries. The genetic ancestry of a patient must be considered when selecting a treatment strategy. Such considerations will be conducive to achieving precision treatment and further improving therapeutic effects and 5-year survival rate.

## Data Availability

Publicly available datasets were analyzed in this study. This data can be found here: https://cancergenome.nih.gov/.

## Abbreviations

OSCC, oral squamous cell carcinoma; TCGA, the cancer genome atlas; HE, hematoxylin–eosin; EUR, primarily European; AFR, African; IARC, international agency for research on cancer; TCPA, the cancer proteome atlas; TIDE, tumor immune dysfunction and exclusion; TSR, tumor–stroma ratio; TILs, tumor-infiltrating lymphocytes; PCA, principal component analysis; ROC, receiver operator characteristic; OS, overall survival; CR, complete remission/response; PFS, progression-free survival; LIHC, liver hepatocellular carcinoma; BRCA, breast invasive carcinoma; COAD, colon adenocarcinoma; OV, ovarian serous cystadenocarcinoma; KIRC, kidney renal clear cell carcinoma; PAAD, pancreatic adenocarcinoma; BLCA, bladder urothelial carcinoma; STAD, stomach adenocarcinoma.
